# KU80 suppresses endonuclease G activity to preserve genomic integrity

**DOI:** 10.1111/febs.70495

**Published:** 2026-03-16

**Authors:** Jargalan Batsaikhan, Kwang‐Hyun Park, Hae Ryung Chang, Eunyoung Jung, Myung‐Jin Kim, Jeeyoon Kang, Hyunjo Shim, Eui‐Jeon Woo, Yonghwan Kim

**Affiliations:** ^1^ Department of Biological Sciences and Research Institute of Women's Health Sookmyung Women's University Seoul Republic of Korea; ^2^ Critical Diseases Diagnostics Convergence Research Center Korea Research Institute of Bioscience & Biotechnology (KRIBB) Daejeon Republic of Korea; ^3^ Department of Proteome Structural Biology, KRIBB School of Bioscience University of Science and Technology (UST) Daejeon Republic of Korea; ^4^ Department of Life Science Handong Global University Pohang Republic of Korea; ^5^ Biodesign & Editing Research Center KRIBB Daejeon Republic of Korea

**Keywords:** endonuclease G (ENDOG), genomic integrity, KU80

## Abstract

Endonuclease G (ENDOG) is involved in DNA replication, mitochondrial DNA maintenance, and late apoptosis. Its role in genome maintenance and cell death necessitates strict regulation to prevent unwanted DNA damage. Although inhibitors of ENDOG have been identified in nonmammalian species, no such inhibitors have been discovered in mammals. Here, we identify KU80 as a novel negative regulator of ENDOG in human cells. The KU80 C‐terminal domain (CTD) shares structural homology with *Drosophila* EndoG inhibitor (EGI), directly binds ENDOG and inhibits its nuclease activity *in vitro*. *In silico* modeling and immunoprecipitation reveal the KU80 core domain mediates ENDOG interaction. Depletion of KU80 increases nuclear ENDOG accumulation and DNA damage under normal conditions, evidenced by elevated phosphorylated histone H2AX (γH2AX) levels. Conversely, ENDOG‐deficient cells show reduced DNA damage and increased chemotherapeutic resistance. We uncover a mechanism where KU80 maintains genomic stability by inhibiting ENDOG activity and nuclear translocation. This study establishes KU80 as a key DNA damage response regulator, offering insights into potential therapies for diseases linked to dysregulated ENDOG activity.

## Graphical abstract

Under normal conditions, EndoG remains restricted to mitochondria and the genome remains intact. When KU80 is absent, EndoG translocates into the nucleus, where it promotes DNA fragmentation and genomic instability. Thus, this work highlights the importance of KU80 in tightly controlling EndoG localization to preserve genome stability.

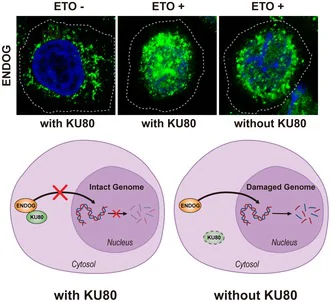

**Abbreviations**
CAD: caspase activated DNase
c‐Myc‐tag: tag derived from myelocytomatosis oncogene
CPT: camptothecin
CTD: C‐terminal domain
DFF: DNA fragmentation factor
DMSO: dimethyl sulfoxide
DSBs: double‐strand breaks
ENDOG: endonuclease G
ETO: etoposide
HA‐tag: epitope tag derived from hemagglutinin
IB: immunoblotting
IF: immunofluorescence
MLS: mitochondrial localization sequence
NHEJ: nonhomologous end joining
siRNA: small‐interfering ribonucleic acid
γH2AX: phosphorylated histone family member X

## Introduction

Genomic stability is fundamental to cellular function and organismal survival [1, 2]. Cells face constant threats to genomic integrity from both intrinsic factors like replication errors and metabolic byproducts, and extrinsic factors, such as ionizing radiation, UV light, and chemical carcinogens [3, 4]. To maintain genomic fidelity, cells have developed sophisticated mechanisms coordinating cellular metabolism, DNA repair, and cell cycle progression. If DNA damage is beyond repair, cells execute programmed cell death. These are coordinated processes, where the cell cycle is paused and apoptosis is inhibited to ensure sufficient time for DNA repair. Endonucleases play pivotal roles in these processes, participating in DNA repair as well as apoptosis. However, their ability to cleave phosphodiester bonds in DNA and RNA necessitates stringent regulation to prevent unintended nucleic acid degradation and maintain cellular homeostasis [5, 6]. Apoptosis, a programmed cell death process, is crucial for normal embryonic development, tissue homeostasis maintenance, and appropriate physiological responses to tissue injury. A hallmark of late‐stage apoptosis is inter‐nucleosomal DNA fragmentation [7]. Upon apoptotic activation, various factors are released from mitochondria, including cytochrome c, procaspases, Smac/DIABLO, apoptosis‐inducing factor (AIF), and Endonuclease G (ENDOG) [8].

ENDOG, a mitochondrial enzyme, translocates to the nucleus in response to apoptotic stimuli, cleaving chromosomal DNA via a caspase‐independent pathway [9, 10]. Initially identified in chicken erythrocyte nuclei, ENDOG efficiently cleaves double‐stranded DNA rich in cytosine and guanine stretches. Synthesized as a 33 kDa precursor protein with a mitochondrial localization sequence (MLS), ENDOG is directed to mitochondria where it is cleaved to produce the active 28 kDa mature nuclease [11, 12]. This mitochondrial compartmentalization under normal conditions prevents indiscriminate nucleic acid degradation [13]. ENDOG belongs to a large family of DNA/RNA nonspecific nucleases characterized by a ββα‐Me‐finger motif, shared among well‐conserved nucleases across microbial and eukaryotic organisms [14]. Under physiological conditions, ENDOG exhibits low nuclease activity and is believed to require additional cofactors for regulation, such as nuclear proteins like AIF, flap endonuclease 1 (FEN‐1), and heat shock protein 70 (Hsp70) [15, 16].

Previous biochemical and structural studies have identified an endogenous ENDOG inhibitor (EGI) in *Drosophila melanogaster*. EGI, localized in the nucleus, protects cells from chromosomal damage by directly binding to the active sites of ENDOG [17, 18]. However, mammalian genomes lack proteins with sequence homology to EGI, suggesting alternative regulatory mechanisms for ENDOG in higher organisms. Comparative structural analyses have suggested that the human protein KU80 (also known as XRCC5) may serve a similar inhibitory function to EGI in mammals [17]. KU80, an 86 kDa protein with three conserved domains, traditionally functions as a heterodimer with KU70 in the nonhomologous end joining (NHEJ) pathway of DNA double‐strand break (DSB) repair [19, 20]. Emerging evidence indicates that KU80 can exist as a monomer in the cytoplasm and may have functions independent of KU70 and DNA repair [21].

In this study, we investigate the potential regulatory role of KU80 on ENDOG activity. We demonstrate that both active and inactive forms of ENDOG interact with the core domain of KU80. Our findings reveal that in the absence of KU80, ENDOG translocates into the nucleus, potentially contributing to DNA degradation. Notably, KU80‐deficient cells exhibit increased DNA damage sensitivity, evidenced by elevated levels of phosphorylated H2AX (γH2AX), whereas ENDOG‐deficient cells show reduced γH2AX intensity. These results suggest that ENDOG is capable of inducing DNA DSB, and that KU80 is the negative regulator of ENDOG. Inhibition is achieved by directly interacting with ENDOG and preventing its nuclear translocation, thereby playing a crucial role in maintaining genomic stability. Our work provides new insights into the regulation mechanism of apoptotic nuclease ENDOG, opening avenues for potential therapeutic interventions in diseases characterized by dysregulated apoptosis and DNA damage responses.

## Results

### KU80 CTD inhibits mammalian ENDOG activity

To identify potential regulators of mammalian ENDOG, we employed a structure‐based approach using the ENDOG–EGI complex as a template (PDB ID: 3ISM). Structural similarity searches conducted with Foldseek revealed that the C‐terminal domain (CTD) of human KU80 (PDB ID: 1Q2Z) exhibits a significant structural resemblance to EGI, achieving a similarity score of 89 and an *E*‐value of 6.35e‐2 (Fig. 1A) [22]. Remarkably, this structural homology exists despite a low sequence identity of only 15.5% between the two proteins. Superimposition of the KU80 CTD onto EGI demonstrated alignment across nearly all regions, including the critical ENDOG binding interface, with a root mean square deviation (RMSD) of 1.941 Å (Fig. 1B). To assess whether the KU80 CTD could inhibit ENDOG activity, we cloned and purified the KU80 CTD encompassing amino acids 590–709. *In vitro* DNase activity inhibition assays were performed using purified ENDOG and varying concentrations of the KU80 CTD, with plasmid DNA serving as the substrate. The results indicated that the KU80 CTD effectively inhibited ENDOG‐mediated DNA cleavage in a dose‐dependent manner, exhibiting inhibitory potency comparable to that of EGI (Fig. 1C). To confirm a direct interaction between ENDOG and the KU80 CTD, we conducted isothermal titration calorimetry (ITC) experiments. The ITC analysis revealed a dissociation constant (*K*_d_) of 78.1 nm, indicative of a high‐affinity binding interaction between the two proteins (Fig. 1D). These findings provide evidence that the KU80 CTD functions as a regulator of ENDOG in mammals, utilizing structural mimicry of EGI to inhibit ENDOG activity despite low sequence conservation.

**Fig. 1:**
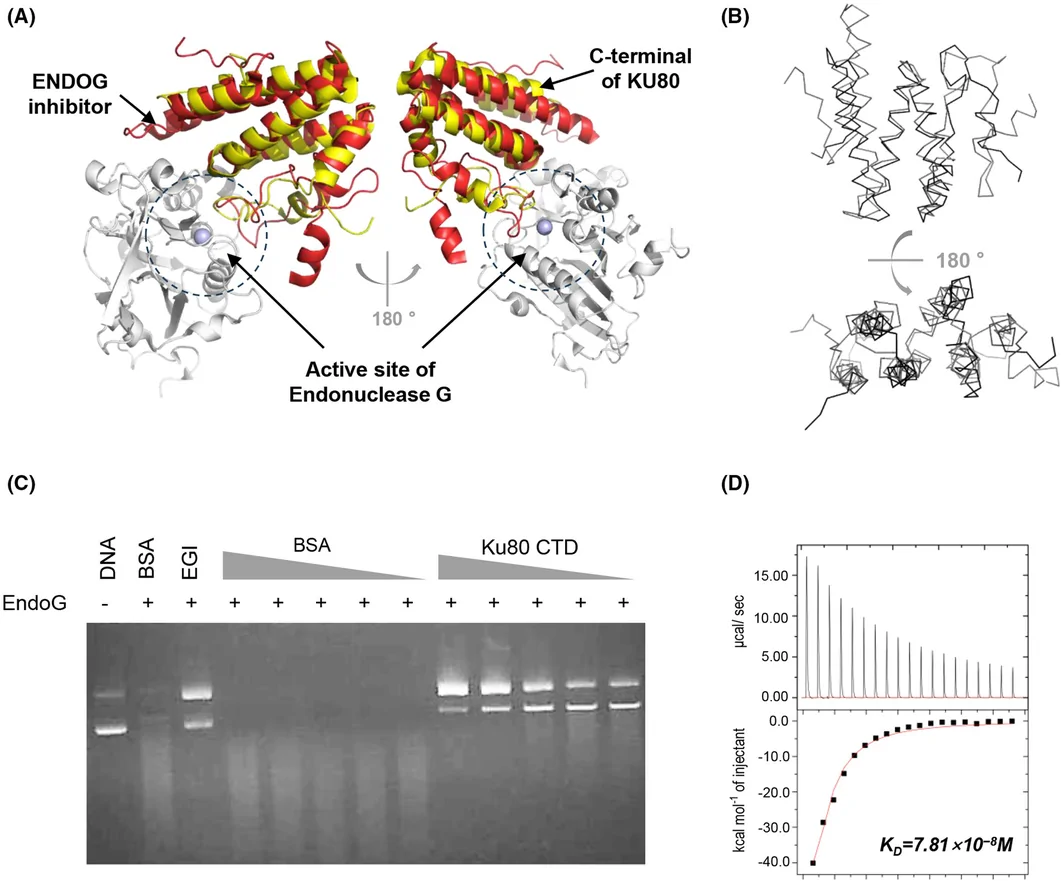
Structural and functional analysis of KU80 C‐terminal domain (CTD) as an endonuclease G (ENDOG) regulator. (A) Structural superposition of the KU80 C‐terminal domain (yellow) with the EndoG inhibitor (red) from the EndoG/EndoG inhibitor (EGI) complex (PDB ID 3ISM). Structural alignment and visualization were performed using PyMOL (Schrödinger, LLC). The active site of ENDOG is depicted in gray, with the magnesium ion cofactor shown as a blue sphere. (B) Ribbon diagram illustrating the backbone alignment between EGI and KU80 CTD, viewed from two orientations rotated 180° around the horizontal axis. (C) DNase activity inhibition assay. Plasmid DNA was incubated with ENDOG (100 nm) and increasing concentrations of either BSA or KU80 CTD (100 nm to 10 μm). EGI (500 nm) served as a positive control. DNA fragmentation was analyzed by agarose gel electrophoresis. (D) Isothermal titration calorimetry (ITC) analysis of the interaction between ENDOG and KU80 CTD. ENDOG (0.01 mm) was titrated with KU80 CTD (1 mm) at 37 °C. The dissociation constant (*K*_d_) was determined to be 78.1 nm.

### *In silico* analysis of ENDOG–KU80 complex

We employed alphafold 3 for *in silico* modeling of the ENDOG–KU80 complex, incorporating UniProt post‐translational modification data to enhance biological relevance [23]. The predicted structure of the ENDOG homodimer complexed with full‐length KU80 revealed specific interaction interfaces (Fig. 2A). The ENDOG‐interacting regions of KU80, comprising the core (residues 236–564) and C‐terminal (residues 565–732) domains, showed predicted local distance difference test (pLDDT) scores of 79.23 and 65.54, respectively, indicating high model confidence (Fig. 2B,C). Interaction energies, quantified using Rosetta with physics‐based force fields and statistical potentials, demonstrated strong binding between full‐length KU80 and ENDOG (−228.7 kcal·mol^−1^) [24]. The KU80 core and C‐terminal regions contributed −111.0 kcal·mol^−1^ and − 66.7 kcal·mol^−1^, respectively. N‐terminal (residues 236–732) and C‐terminal (residues 1–590) truncations yielded interaction energies of −177.2 kcal·mol^−1^ and −110.3 kcal·mol^−1^, respectively (Fig. 2D). These results identify the KU80 core region as the primary ENDOG interaction domain, with significant contributions from the C‐terminal region.

**Fig. 2:**
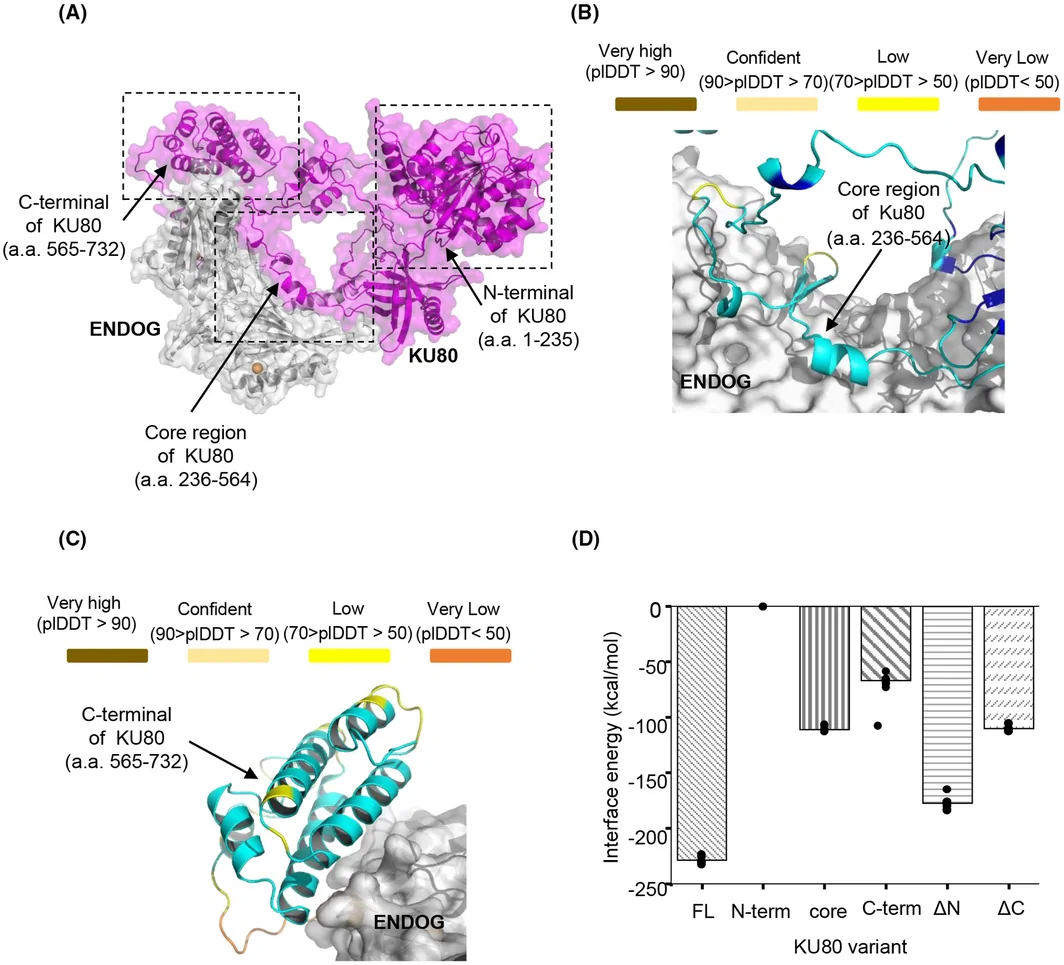
Complex model showing endonuclease G (ENDOG)–KU80 interaction. (A) AlphaFold 3‐predicted structure of the ENDOG homodimer (gray) with full‐length KU80 (magenta). Key KU80 regions are highlighted in red boxes: N‐terminal (residues 1–235), core (residues 236–564), and C‐terminal (residues 565–732). Structural visualization and rendering were performed using pymol (Schrödinger, LLC). (B) Detailed view of the KU80 core region (cartoon) interacting with ENDOG (surface). AlphaFold model confidence: very high [dark blue, predicted local distance difference test (pLDDT) greater than 90], confident (light blue, pLDDT 70–90), low (yellow, pLDDT 50–70), very low (orange, pLDDT less than 50). (C) C‐terminal region of KU80 interacting with ENDOG, confidence scores as in B. (D) Thermodynamic interface energies of the complex model using Rosetta REF2015 force field. Energies are shown for full‐length KU80 (FL), N‐terminal (residues 1–235, N‐term), core (residues 236–564), C‐terminal (residues 565–732, C‐term), N‐truncation (residues 235–732, ΔN), and C‐truncation (residues 1–590, ΔC). Each variant is represented by a bar showing the average energy, with individual dots indicating the energies of 10 different relaxed models per variant.

### KU80 interacts with the ENDOG through its core domain

Because the nuclease activity of ENDOG was suppressed by KU80 and *in silico* analysis elucidated the protein–protein complex, we next decided to directly validate the interaction between ENDOG and KU80 using an IP assay and determine the critical domain for this interaction. First, the mutagenesis plasmids *ENDOG* and *KU80* were used to clone the truncation mutants of each protein. Afterward, a co‐immunoprecipitation (Co‐IP) assay was conducted to identify the essential domains for interaction (Fig. 3A). The plasmids were labeled with HA and Myc tags to facilitate the specific isolation of proteins of interest from protein complexes. Co‐IP assay revealed that both the mature and preprotein forms of ENDOG were associated with KU80 (Fig. 3B). Additionally, the interaction between endogenous ENDOG and KU80 was confirmed (Fig. S1A,B). Next, we investigated which KU80 domain was critical for its interaction with ENDOG. It has been demonstrated that KU80 has three main active domains, including an N‐terminal, von Willebrand factor type A (vWA) domain (1–235 aa), a core domain (236–564 aa), and a C‐terminal domain (565–732 aa). The vWA domain is responsible for DNA binding, whereas the core domain mediates protein–protein interactions [20, 25]. Co‐IP results demonstrated that the core domain of KU80 played a critical role in this interaction (Fig. 3C). This result was supported by an additional IP assay with deletion mutants of KU80 and full‐length ENDOG. All deletion mutants interacted with ENDOG, except for the core domain deletion mutant (Fig. 3D). As the previous results revealed a direct interaction between ENDOG and KU80, the next step was to investigate the specific mechanism of this interaction. Previous studies have reported that ENDOG translocates to the nucleus after being released from the mitochondria during apoptosis and contributes to nuclear DNA fragmentation [12, 26]. To replicate this scenario, cells were treated with ETO to induce DNA damage, which induced apoptosis via DNA DSBs [27, 28]. Cells were transfected with *ENDOG*‐ or *KU80*‐targeting siRNAs and treated with ETO for the indicated times. Remarkably, IB analysis revealed that ENDOG and KU80 deficiency did not exert discernible effects on the reciprocal protein expression. No significant differences were observed in KU80 expression between ENDOG‐depleted cells under normal and apoptotic conditions (Fig. 3E). Correspondingly, ENDOG expression did not change in the absence of KU80 under nonapoptotic or ETO‐treated conditions (Fig. 3F). Taken together, these data suggest that both the full‐length and mature forms of ENDOG are associated with KU80 through its core domain of KU80, and neither ENDOG nor KU80 drastically affects the expression of the reciprocal protein.

**Fig. 3:**
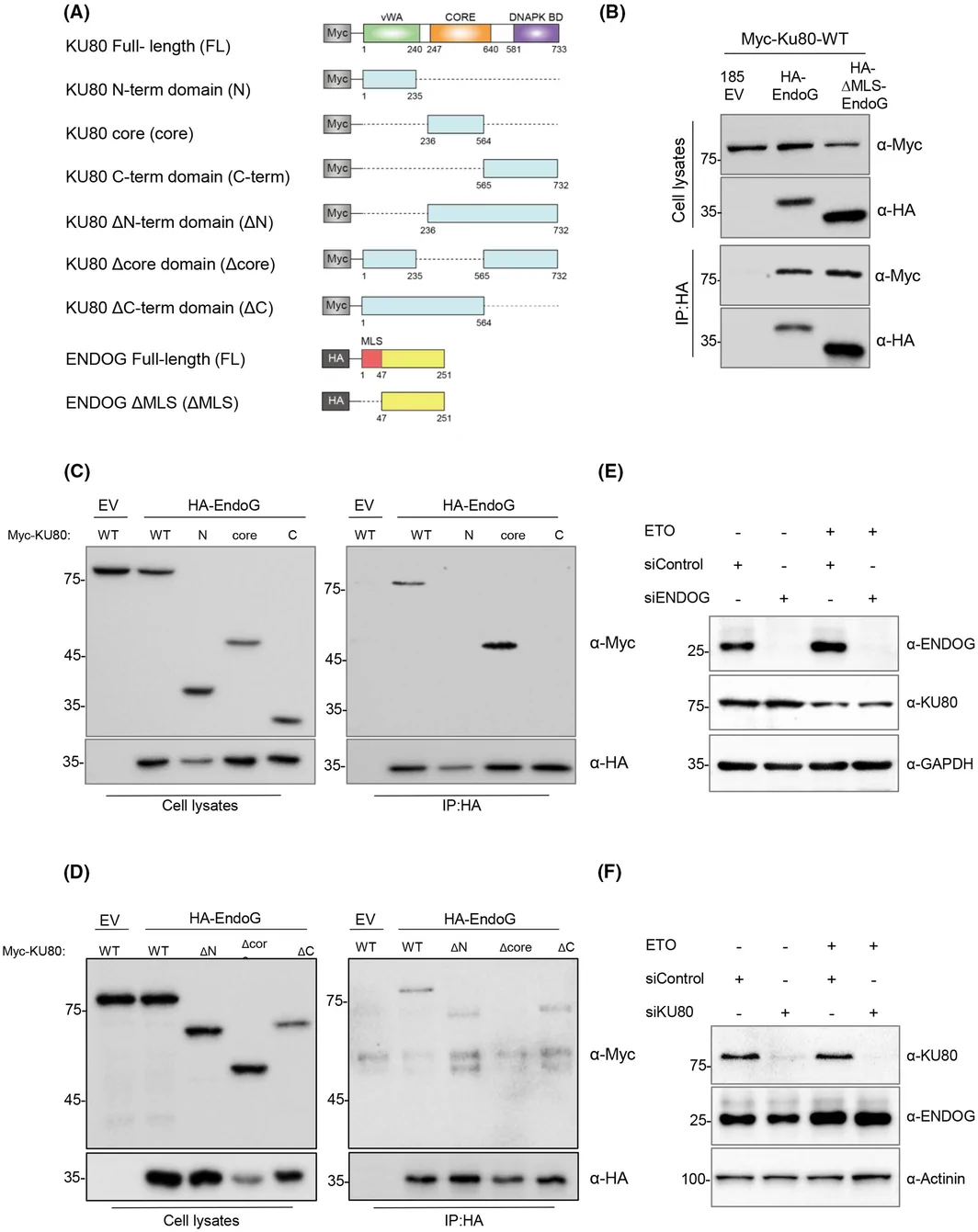
KU80 is associated with the endonuclease G (ENDOG) through its core domain, and neither protein affects the expression of the other. Human HEK‐293T and Hela cells were used. (A) A mapping graph presenting the recombinant variants of KU80 and ENDOG. (B) Co‐immunoprecipitation (Co‐IP) results showed that HA‐ENDOG and HA‐ΔMLS‐ENDOG interacted with KU80 (C) Co‐IP analysis revealed the interaction between the core domain (aa 236–564) of KU80 and full‐length ENDOG. (D) Co‐IP analysis demonstrated the inability of core domain deletion mutants of KU80 to associate with ENDOG. (E) The deficiency of ENDOG did not affect the expression of KU80 protein under normal and apoptotic conditions. (F) The immunoblot image illustrated that the protein level of ENDOG was not modified in the absence of KU80 in Hela cells under normal and apoptotic conditions.

### KU80 absence leads to ENDOG accumulation within the nucleus

KU80 localizes mostly in the nucleus and functions as a heterodimer. However, KU80 localizes not only in the nucleus but also in the cytosol as a monomer [19, 21]. Although minimal amounts are present in the nucleus, the majority of ENDOG generally resides in the mitochondrial intermembrane space under normal conditions and is translocated to the nucleus during late apoptosis [12, 26, 29]. An immunofluorescence (IF) assay was performed to validate the localization of these two proteins in HeLa cells. Interestingly, ENDOG localized to the nucleus dramatically increased in the absence of KU80 (Fig. 4A). Statistical analysis demonstrated that the ENDOG intensity per nucleus was significantly enhanced in KU80‐deficient cells (Fig. 4B,C). Collectively, the IF assay results demonstrated that an increased amount of ENDOG was translocated to the nucleus in the absence of KU80 under nonapoptotic conditions, implying that ENDOG is capable of nuclear localization in the absence of a negative regulator.

**Fig. 4:**
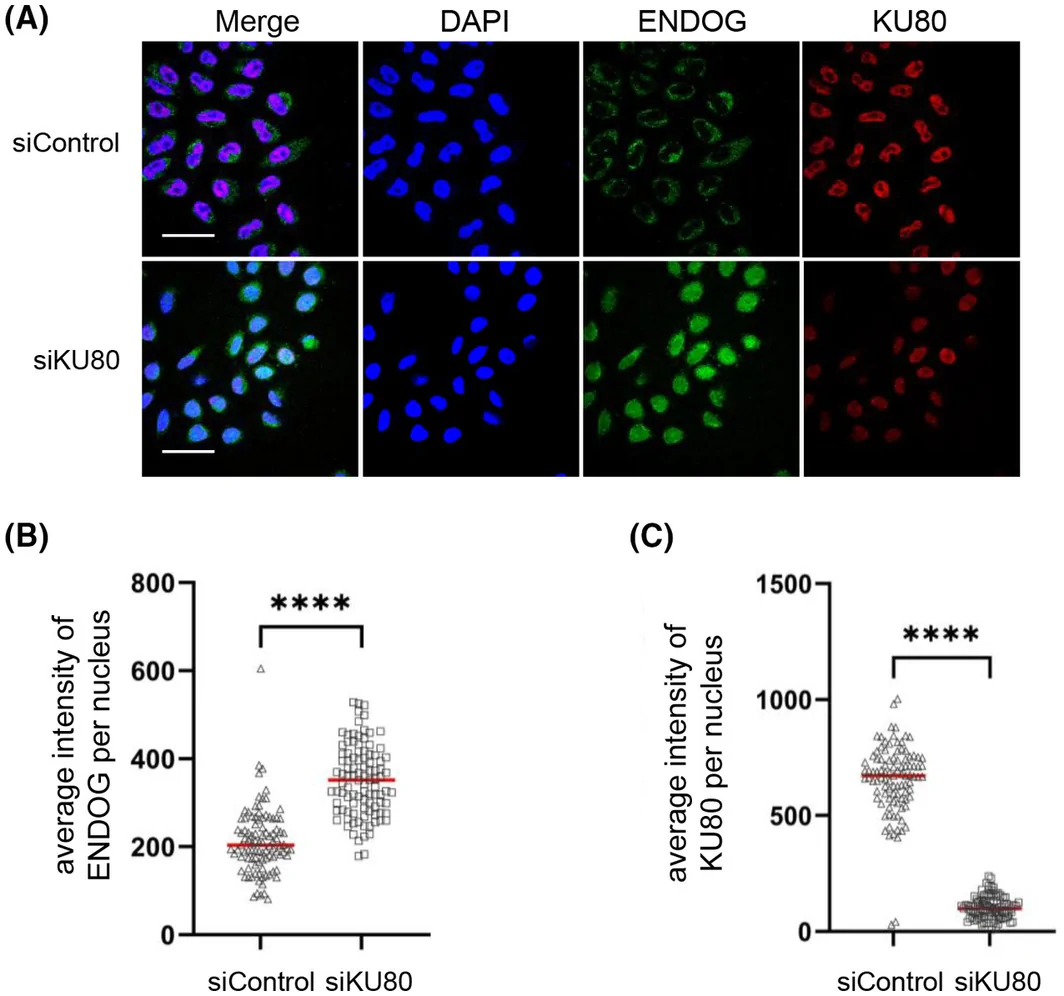
Absence of KU80 induces increased endonuclease G (ENDOG) recruitment to the nucleus. (A) Immunofluorescent images for the accumulation of ENDOG (green) in the nucleus in KU80‐deficient or control human HeLa cells. The cells were treated with KU80/XRCC5 targeting siRNA and control siRNA, fixed, and permeabilized (scale bar, 50 μm). (B, C) Representative bar charts illustrating the intensity of ENDOG or KU80 per nucleus. Each dot represents the average nuclear intensity of an individual cell, with the red horizontal line indicating the median value. Statistical significance was determined using an unpaired, two‐tailed Student's *t*‐test (****P < 0.0001).

ENDOG is known to translocate into the nucleus during apoptosis. To exclude the possibility that the nuclear accumulation of ENDOG observed after KU80 depletion simply reflects apoptosis, we assessed cell death under the same experimental conditions. As shown in Fig. S3A,B, KU80 depletion did not increase apoptotic cells compared with control. These results indicate that the nuclear translocation of ENDOG in KU80‐depleted cells does not arise from apoptosis but is specifically attributable to the loss of KU80.

### ENDOG accumulation in the nucleus induces DNA damage response

The above results suggest that ENDOG can generate DSBs in the nuclear DNA when it translocates to the nucleus in the absence of KU80 through its sugar nonspecific nuclease activity. Nuclear ENDOG expression is spatially correlated with apoptotic DNA fragmentation [30]. The most common and accurate approach to assess apoptosis and DNA DSBs is to measure the phosphorylated variant of the histone, known as γH2AX, as a biomarker due to its formation in the initial stages of DNA DSB occurrence [31, 32]. Furthermore, the presence of γH2AX is a common feature of apoptosis, which is attributed to its formation during ETO‐induced apoptosis [31]. Interestingly, IF images demonstrated a weak signal of γH2AX (red) in the absence of KU80 under normal conditions (Fig. 5A), and simultaneously the accumulation of ENDOG drastically increased in the nucleus in cells lacking KU80 (Fig. 5A, middle panel). Representative graphs demonstrated a significant difference in the intensity of γH2AX between control and ETO‐treated cells (Fig. 5B,C). However, the intensity of γH2AX decreased even under apoptotic conditions in ENDOG‐deficient cells (Fig. 5D). In addition, ENDOG accumulated more in the nucleus compared with normal condition and under ETO‐induced apoptotic conditions (Fig. 5D and Fig. S2). Quantitative analysis of ENDOG and γH2AX intensity per nucleus confirmed the reduction of γH2AX under *ENDOG* knockdown conditions (Fig. 5E,F). Further study revealed that the overexpression of KU80 significantly impaired damage‐induced ENDOG translocation to the nucleus (Fig. S4), suggesting an inhibitory role of KU80 in ENDOG translocation. This confirms that ENDOG is capable of accumulating in the nucleus and inducing DSB even in nonapoptotic conditions. This implies that a negative regulator is necessary to inhibit ENDOG during normal conditions. Collectively, these results reveal KU80 inhibits ENDOG, as indicated by its localization and γH2AX expression level.

**Fig. 5:**
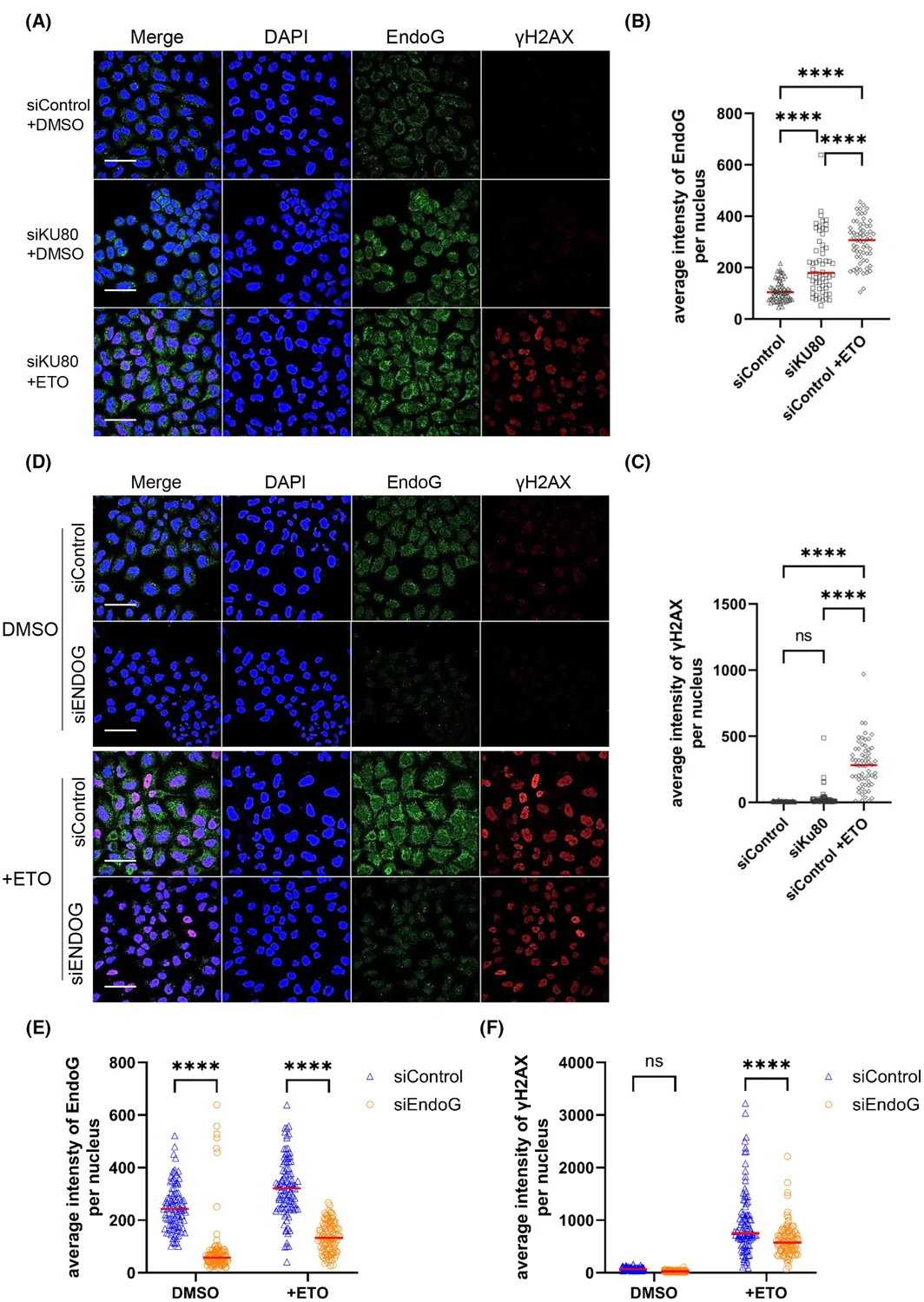
Accumulation of endonuclease G (ENDOG) in the nucleus induces DNA damage response. (A) KU80‐deficient cells encounter an enhanced level of DNA damage, as evident from γ‐H2AX (red) levels, and showed elevated accumulation of ENDOG (green) within the nucleus in the absence of KU80 and etoposide‐treated Hela cells. Scale bars for all panels represent 50 μm. (B, C) Graphs (B and C) represent the quantification of the data shown in (A). Bar charts for quantifying the average intensity of ENDOG and γ‐H2AX per nucleus. Each dot represents the average nuclear intensity of an individual cell, with the red horizontal line indicating the median value. Statistical significance was determined using one‐way ANOVA followed by Tukey's multiple comparisons test (****P < 0.0001). (D) The intensity of γ‐H2AX (red) is decreased in ENDOG (green)‐deficient cells. (E, F) Quantification of nuclear ENDOG and γ‐H2AX signal intensities. Each dot represents the average nuclear intensity of an individual cell, with the red horizontal line indicating the median value. Statistical significance was determined using two‐way ANOVA followed by Sidak's multiple comparisons test (****P < 0.0001). siControl and siENDOG were transfected as indicated. Cells were treated with 50 μm etoposide for 16 h to induce apoptosis.

### ENDOG deficiency suppresses the sensitivity of cells to chemotherapeutic agents, including etoposide and camptothecin

We have shown that ENDOG can induce DNA DSBs after its translocation to the nucleus in Fig. 5. We hypothesized that ENDOG‐deficient cells would be more resistant to chemotherapeutic agents compared with control cells, as we have observed decreased levels of γH2AX after ETO treatment when *ENDOG* was knocked down (Fig. 5D). Following siRNA‐mediated *ENDOG* silencing, cells were treated with the indicated concentrations of ETO and CPT. Subsequently, the cells were incubated for 10 days to allow colony formation. Colony quantification demonstrated that ENDOG‐deficient cells were more resistant to high concentrations of ETO and CPT (Fig. 6A,B). Quantitative analysis revealed that ENDOG deficiency enhanced colony formation (Fig. 6C,D). Taken together, these data suggest that cells lacking ENDOG are more resistant to chemotherapeutic agents, such as ETO and CPT.

**Fig. 6:**
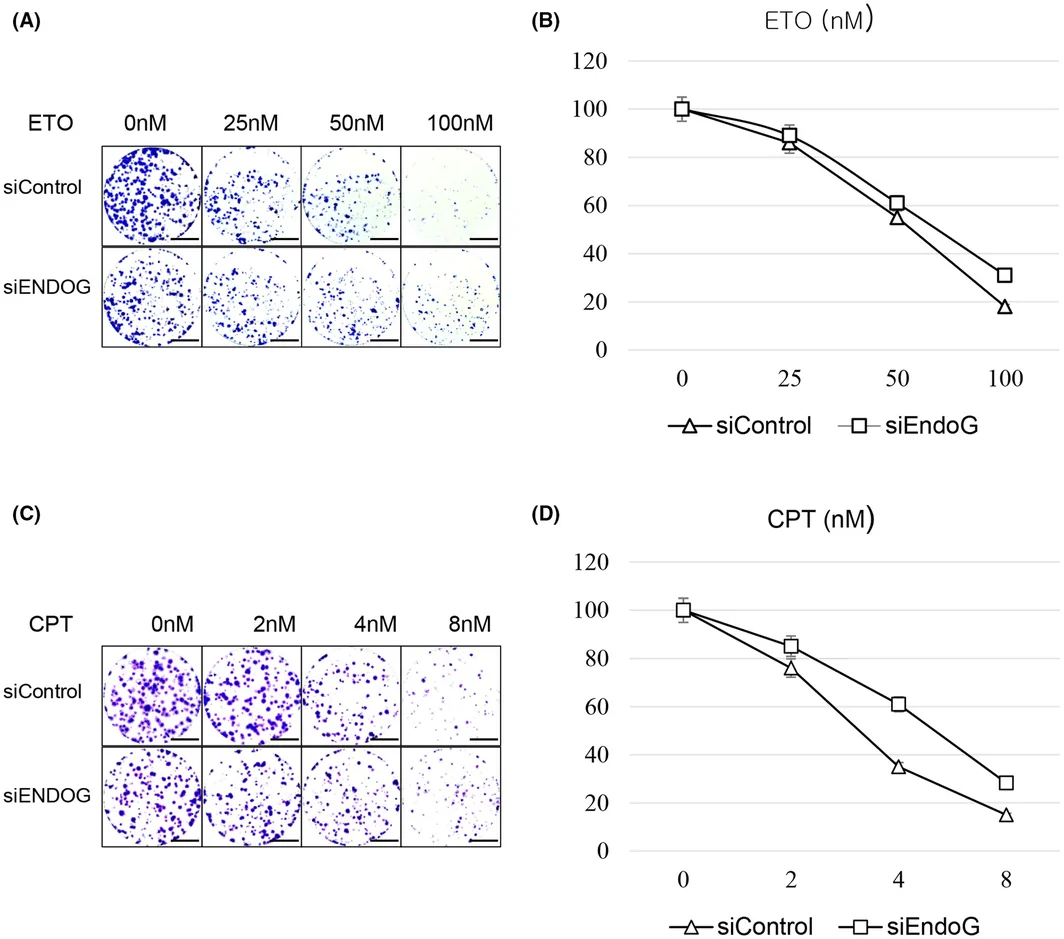
Endonuclease G (ENDOG)‐deficient Hela cells are resistant to etoposide and camptothecin. (A) Representative images for clonogenic assays (scale bar, 1 cm). The cells were transiently transfected with nontargeting and ENDOG‐targeted siRNAs and treated with 25, 50, and 100 nm etoposide and vehicle (DMSO) only for control on subsequent days. (B) The graph shows a comparison of control and ENDOG‐deficient cells forming colonies at indicated concentrations of etoposide (mean ± SD; *n* = 3 biologically independent replicates). (C) Representative images for clonogenic assays using camptothecin at concentrations of 2, 4, and 8 nm and only vehicle for control (scale bar, 1 cm). (D) The graph illustrates the contrast between colonies formed by control cells and ENDOG‐deficient cells at specified concentrations of camptothecin (mean ± SD; *n* = 3 biologically independent replicates).

## Discussion

Endonuclease regulation is critical for cell viability and apoptosis. Despite the recognized role of ENDOG in apoptotic DNA degradation in mammalian cells, its regulatory mechanisms remain incompletely characterized. Here, we reveal a direct interaction between mammalian ENDOG and KU80, uncovering a novel inhibitory function of KU80. The CTD of KU80 directly inhibits ENDOG activity, while the core domain mediates protein interaction. This finding expands the functional repertoire of KU80 beyond its established role in NHEJ DNA repair. Structural analysis revealed that the solenoid domain of KU80 exhibits remarkable homology to *Drosophila* EGI, with comprehensive domain searches identifying KU80 (but not KU70, DNA‐PKcs, XRCC4, or Ligase IV) as the sole mammalian protein structurally analogous to EGI, demonstrating comparable inhibitory potency against ENDOG. Although the KU80 CTD shares a very low sequence identity of just 15.5% with the *Drosophila* EGI, it exhibits a highly conserved structural topology that aligns almost perfectly across the critical ENDOG binding interface [17, 18]. Functionally, this structural mimicry allows the KU80 CTD to perform an equivalent regulatory role to EGI, directly binding to ENDOG and suppressing its nuclease activity with comparable dose‐dependent potency. Thus, despite their phylogenetic divergence in primary sequence, the striking structural and functional parallels between the KU80 CTD and EGI highlight a conserved mechanism for restraining apoptotic nucleases to preserve genomic integrity. Furthermore, *in silico* modeling and immunoprecipitation experiments revealed that the KU80 core domain also plays an essential role in ENDOG interaction.

Our findings suggest that KU80 regulates ENDOG through direct protein–protein interactions, not by altering its expression level. This regulatory relationship represents a previously unrecognized but functionally conserved mechanism for genome protection, with KU80 serving as the mammalian guardian against aberrant ENDOG activity, functionally equivalent to Drosophila EGI. Our data highlight ENDOG inhibition by KU80 mainly in the nucleus, as shown by the increased accumulation of ENDOG and decreased γH2AX. Based on the control condition, KU80 most likely sequesters ENDOG in the cytosol, preventing nuclear translocation and subsequent DNA degradation. This is because a very little to undetectable amount of ENDOG is normally present in the cytosol or in the nucleus as shown in Figs 4 and 5. Knock down of *KU80* dramatically increasing the nuclear localization of ENDOG strongly implies that there must be a basal level inhibition of its migration to the nucleus which involves KU80. This cytosolic inhibition is crucial given the nucleic acid cleavage activity of ENDOG and its role in mitochondrial DNA replication primer provision.

Our findings support a coordinated mechanism whereby KU80 maintains genome integrity by simultaneously promoting DNA repair and restraining apoptosis. While our *in vitro* DNase inhibition assays and ITC experiments demonstrated that isolated KU80 CTD directly binds to ENDOG and inhibits its nuclease activity, Co‐IP experiments in cellular contexts failed to detect CTD‐ENDOG interaction when CTD was expressed alone. This apparent discrepancy likely reflects the requirement for proper protein context and cellular localization signals present in full‐length KU80 but absent in isolated CTD, suggesting that while CTD harbors the intrinsic ENDOG‐inhibitory function, its effective engagement *in vivo* depends on structural elements provided by the complete KU80 protein. These observations underscore the importance of considering both domain‐intrinsic biochemical capabilities and cellular contextual requirements when interpreting protein–protein interaction data. Under sub‐lethal genotoxic stress, KU80 functions as a KU70/KU80 heterodimer positioned predominantly in the nucleus, rapidly binding to DNA breaks and facilitating classical NHEJ. This ‘repair‐first’ bias is reinforced by KU‐dependent suppression of apoptotic signaling. KU binds to a stem loop in the p53 mRNA 5′UTR under normal conditions to repress p53 translation, thereby preventing unnecessary p53‐mediated cell death when damage is repairable [33]. KU80‐deficient mammalian cells exhibited increased ENDOG nuclear translocation, leading to DNA damage evidenced by elevated γH2AX levels. Conversely, ENDOG‐deficient cells showed decreased γH2AX levels under normal and apoptotic conditions, with increased chemotherapeutic resistance.

However, under severe or sustained DNA damage conditions, KU80 undergoes stress‐triggered modifications and relocalization that shift its role from ‘guardian’ to ‘executioner’. High‐intensity stress or prolonged DNA damage activates post‐translational modifications on KU80 and KU70 that attenuate the repair regime. Upon significant genotoxic stress, KU70 becomes acetylated, disrupting its interaction with the pro‐apoptotic factor Bax. More definitively, proteolytic cleavage of KU80 represents an early event in apoptosis. KU80 is cleaved at its C terminus by caspase/calpain activity in the nucleus, disabling the DNA‐PK‐KU complex [34]. The cleaved KU80 fragments redistribute to the cytoplasm as the nucleus fragments. This loss of intact KU80 not only halts NHEJ repair but also releases the restraint on apoptotic nucleases. These findings align with previous observations of ENDOG activation through subcellular translocation during late apoptosis in mammalian systems. In Drosophila, apoptosis induction reduces and relocalizes inhibitor levels to the cytoplasm. Similarly, mammalian KU80 undergoes cleavage and cytoplasmic redistribution during early apoptosis. Notably, ENDOG nuclear translocation under normal conditions in KU80‐absent cells suggests a regulatory role for KU80 in mammalian ENDOG activation.

This study uncovers a novel regulatory mechanism critical for genome maintenance and cell survival in mammalian systems. Structural insights into the ENDOG–KU80 complex suggest a regulatory function for cytosolic monomeric KU80. Our proposed working model positions KU80 as a ‘fidelity checkpoint’ that operates based on damage levels, cellular state, and molecular modifications. KU80 functions as a molecular switch that prioritizes repair under mild stress but capitulates to apoptosis under overwhelming damage. This model explains how KU80 ‘divides its allegiance’ between DNA repair and apoptosis, demonstrating its role as a sophisticated quality control system for genomic integrity. Future research should elucidate the *in vivo* significance of KU80‐ENDOG interaction across mammalian models, explore potential cancer therapeutic applications, and investigate similar regulatory mechanisms in other apoptosis and DNA repair nucleases. Understanding these interactions will provide valuable insights into diseases associated with DNA damage and apoptosis dysregulation, advancing knowledge of mammalian genome maintenance and cell viability. In conclusion, this study identifies KU80 as a critical regulator of ENDOG, providing new insights into genome maintenance and potential therapeutic approaches for diseases characterized by dysregulated apoptosis and DNA damage.

## Materials and methods

### Expression and purification of proteins

ENDOG (residues 44–293; NP_031957.1) and KU80 C‐terminal domain (residues 590–709; NP_066964.1) were expressed in *Escherichia coli* BL21 (DE3). ENDOG was cloned into pMAL‐c2X vector and KU80 into pET28a vector. Cultures were grown at 37 °C to OD600 0.7, induced with 0.5 mm IPTG, and incubated at 18 °C for 18 h. For ENDOG, cells were lysed in 30 mm sodium phosphate pH 7.5, 50 mm NaCl, 1 mm EDTA, 10 mm β‐mercaptoethanol. MBP‐ENDOG was purified by amylose affinity chromatography (Cytiva, Marlborough, MA, USA), eluted with 20 mm maltose, and cleaved with TEV protease. Further purification used Mono Q and HiTrap Heparin chromatography (Cytiva). ENDOG was dialyzed into 30 mm Tris–HCl pH 8.0, 3 mm MgCl_2_, 1 mm DTT and concentrated to 8 mg·mL^−1^. For KU80, cells were lysed in 250 mm NaCl, 50 mm HEPES pH 7.5, 5 mm β‐mercaptoethanol, 5% glycerol. His‐KU80 (86) was purified by nickel affinity chromatography, eluted with 500 mm imidazole, and further purified by Superdex 200 size‐exclusion chromatography (Cytiva). The protein was concentrated to 10 mg·mL^−1^ in 500 mm NaCl, 30 mm HEPES pH 7.5, 3 mm β‐mercaptoethanol, 5% glycerol.

### DNase activity inhibition assay

ENDOG (75 nm) was preincubated with KU80 (10–160 μm) in 50 mm Tris–HCl pH 7.5, 50 mm NaCl, 5 mm MgCl₂ at 37 °C for 30 min. Nuclease reaction was initiated by adding 200 ng plasmid DNA (5.6 kb) and incubated for 10 min at 37 °C. Reactions were stopped with 10 mm EDTA and 1× DNA loading dye. Products were separated on 1% agarose gel in TAE buffer at 100 V for 1 h. Gels were stained with 0.5 μg·mL^−1^ ethidium bromide and imaged using a Gel Doc system. Band intensities were quantified using image lab software (Bio‐Rad, Hercules, USA). ENDOG inhibitor (500 nm) served as positive control. BSA at equivalent concentrations to KU80 was used as negative control.

### Structural modeling and Interface analysis

ENDOG–KU80 complex models were generated using alphafold v3. Top models were selected based on pLDDT scores and predicted interface confidence. Models were refined using Rosetta relax protocol with constraints to maintain AlphaFold‐predicted fold. Rosetta InterfaceAnalyzer was used to calculate interface properties, including Δ*G*_interface, solvation energy, hydrogen bonds, salt bridges, and buried surface area. Interface stability was assessed using interface delta score (ΔΔ*G*). The final model was selected based on lowest ΔΔ*G*. Structural analysis was performed using pymol v3 (Schrödinger).

### Cell cultures and treatments

The human cell lines HeLa (RRID:CVCL_0030) and HEK‐293T (RRID:CVCL_0063) were purchased from the American Type Culture Collection (ATCC, Manassas, VA, USA). All cell lines used in this study were authenticated within the past 3 years using Short Tandem Repeat (STR) profiling. For cells purchased directly from ATCC (Manassas, VA, USA), identity was guaranteed by the supplier's certification. Cells were cultured in Dulbecco's modified Eagle's medium (with 4.5 g·L^−1^ D‐glucose and L‐glutamine; Gibco, Grand Island, NY, USA) supplemented with 10% fetal bovine serum and 1% penicillin (10 000 units·mL^−1^) and streptomycin (10 000 μg·mL^−1^) (Gibco). Cells were incubated at 37 °C in a humidified atmosphere with 5% CO_2_. All experiments were performed with mycoplasma‐free cells, as confirmed by routine testing using PCR‐based detection. Camptothecin (CPT) and etoposide (ETO) were used to induce DNA damage and perform clonogenic assays, respectively.

### DNA plasmid transfection

The TransIT‐LT1 (Mirus Bio, Madison, WI, USA) transfection reagent was used for DNA plasmid transfection. Transfection was performed according to the manufacturer's instructions. Briefly, 24 h before plasmid transfection, 1 × 10^6^ HEK‐293 T cells were seeded in a 60‐mm plate. The next day, when cellular confluency reached 80% or more, the plasmids were transiently co‐transfected using the TransIT‐LT1 (Mirus Bio) transfection reagent. The cells were harvested by trypsinization 24 h post‐transfection and prepared for subsequent experiments.

### Co‐immunoprecipitation assay

HA‐ and Myc‐tagged DNA plasmids were used for Co‐IP assays. After 24 h of transfection, the plasmid‐transfected HEK‐293 T cells were trypsinized and rinsed once with Dulbecco's phosphate‐buffered saline (DPBS). Afterward, the cells were lysed using mammalian cell lysis buffer (MCLB: 50 mm Tris, 150 mm NaCl, and 0.5% Nonidet P‐40) containing a protease inhibitor (Roche Diagnostics, Mannheim, Germany) and sonicated thrice for 10 s using an ultrasonic processor KSS‐650D machine at 20‐s intervals and 20% intensity on ice. After sonication, cells were gently rotated at 11 rpm for 30 min at 4 °C. After agitation, the cells were centrifuged at 21 206 ***g*** for 20 min. Supernatants were analyzed by the Bradford assay to measure the protein amount and were separated into two batches: input and immunoprecipitation (IP) samples. In the IP sample, 10 μL of monoclonal anti‐HA resin beads (Sigma‐Aldrich Solutions; Merck, Darmstadt, Germany) was added to pull down the target protein [35]. Afterward, the samples were incubated for 2 h at room temperature or 4 °C overnight with agitation at 11 rpm. Next, the samples were washed five times with the MCLB solution. One wash step involved centrifuging for 5 min at 377 ***g*** at 4 °C, followed by agitation at 4 °C for another 5 min. Following the IP wash, all washing solutions were disposed of completely, and 2× SDS sample buffer (RBC) was added to the samples. The samples were boiled at 100 °C for 10 min to denature the proteins and proceed for immunoblotting (IB). List of antibodies used in these assays are found in Table S1.

### Small‐interfering RNA (siRNA)‐mediated gene silencing

For siRNA‐mediated gene knockdown, human HeLa cells were seeded at a density of 1–5 × 10^5^ cells per 60‐mm plate or 6‐well plate in an antibiotic‐free medium. Reverse transfection of siRNA was performed using Lipofectamine RNAiMAX (Invitrogen, Carlsbad, CA, USA; Thermo Fisher Scientific, Newark, CA, Waltham, MA, USA) concurrently with cell seeding, following the manufacturer's protocol. The following day, forward transfection of siRNA was performed to enhance the efficacy of siRNA transfection [36]. Twenty‐four to forty‐eight hours after forward transfection, the cells were harvested for subsequent experiments, including IF and IB. Negative control siRNA (Invitrogen, Thermo Fisher Scientific) was used as a negative control. siRNA sequence for *ENDOG* and *KU80* is found in Table S2.

### Immunofluorescence assay

A total of 1–1.5 × 10^5^ human HeLa cells were plated in a 6‐well plate with 10‐mm cover glasses. siRNA‐mediated gene depletion was performed using the siRNA transfection method as described previously. Following a transfection period of 72 h, the cells were rinsed twice with 2 mL of cold 1× PBS, followed by fixing with ice‐cold 3.7% formaldehyde for 10 min. After fixation, the cells were washed twice with 1× PBS solution. For cell permeabilization, 0.5% NP‐40 solution was used and incubated for 5 min at room temperature. The cells were washed twice with 1× PBS solution again. Before incubation with primary antibodies, the cells were blocked with 1× PBG solution (0.5% bovine albumin fraction and 0.2% fish gelatin in 1× PBS) for 1 h at room temperature. After blocking, the cells were incubated with the primary antibody at 4 °C overnight [37, 38]. The next day, the cells were washed with 1× PBG solution for 10 min each and probed with fluorophore‐conjugated secondary antibodies for 1 h at room temperature. After secondary antibody incubation, the cells were mounted using the VECTASHIELD^®^ Antifade Mounting Medium with DAPI (Vector Laboratories, Newark, CA, USA) to preserve fluorescence. Images were obtained using a confocal microscope (Nikon A1; Tokyo, Japan, and OLYMPUS FV10i at Gyeongnam Bio and Anti‐aging Core Facility Center) using ×60 oil objective. All foci count quantification was performed using imagej. The list of antibodies used in this assay is found in Table S1.

### Immunoblotting

The collected cell pellets were lysed using the MCLB medium supplemented with a protease inhibitor (Roche Diagnostics). Subsequently, the pellets were sonicated using an ultrasonic processor (KSS‐650D) for two cycles of 10‐s bursts with 20‐s intervals at 20% intensity on ice. Following sonication, the samples were agitated for 30 min at 4 °C, followed by centrifugation at 21 206 ***g*** for 20 min. The resulting supernatants were subjected to Bradford assay to determine protein concentrations. Next, the cell lysates were buffered with sample buffer and boiled at 100 °C for 10 min. The denatured samples were separated on 10 to 12% SDS/PAGE gels and blotted onto a 0.45 μm pore immunoglobin‐P transfer membrane (Merck, Germany) at 30 V and 4 °C overnight [39]. After transfer, the membrane was blocked with a 5% skim milk solution (skim milk powder and Tween 20 in PBS) for 1 h at room temperature and rinsed with 1× PBST (Tween 20 in PBS) for a short time. Next, the primary antibody was added to the membrane with 3% BSA or 5% skim milk and incubated for 2 h at room temperature with gentle rotation. Subsequently, the primary antibodies were completely removed and blots were washed with 1× PBST for 15 min each. The membrane was next incubated with secondary antibodies diluted in 5% skim milk dissolved in 1× PBST and incubated for 1 h at room temperature. After incubation, the unattached antibodies were washed away with 1× PBST for 10 min and membranes were developed using the Lumi Femto or Pico reagent (Daeil Lab Service, Seoul, South Korea). The blots were visualized using a Molecular Imager XR+ System (Bio‐Rad). The list of antibodies used in this assay are found in Table S2.

### Drug sensitivity assays/cell clonogenic assay

A total of 1 × 10^3^ HeLa cells were seeded in 6‐well plates 24 h before drug treatment. The cells were treated with ETO and CPT at concentrations of 0, 25, 50, 100 nm and 0, 2, 4, and 8 nm, respectively, for 10 days to allow colony formation. The cells were rinsed once with ice‐cold 1× PBS and fixed with a 3.7% formaldehyde solution for 15 min. After fixation, the cells were rinsed with 1× PBS again and stained with 0.1% crystal violet solution (100% ethanol) for 15 min, followed by the removal of the crystal violet solution with 1× PBS. The cell colonies were air‐dried overnight. Colonies were measured using the imagej software.

### Data analysis

imagej and prism 10 (prism pad) software were applied to analyze the data and generate graphs for IF and clonogenic survival assays. The statistical analysis was performed by Student's *t*‐test and one‐ and two‐way analysis of variance test using the prism 10 software. Data are presented as median or mean ± standard deviation (ns = nonsignificant >0.05, *P < 0.05, **P < 0.01, ***P < 0.001, ****P < 0.0001).

## Author contributions

YK and E‐JW designed and supervised the study. JB, K‐HP, M‐JK, and JK performed the experiments, analyzed the data, and wrote the manuscript. JB, HC, E‐JW, and YK wrote the manuscript.

## Conflict of interest

The authors declare no conflict of interest.

## Supporting information


**Fig. S1.** Interaction between ENDOG and KU80 detected by endogenous immunoprecipitation in HeLa cells.
**Fig. S2.** Immunofluorescence analysis of ENDOG and KU80 localization in HeLa cells under untreated and etoposide‐treated conditions.
**Fig. S3.** Analysis of apoptotic induction following KU80 depletion.
**Fig. S4.** Immunofluorescence analysis of ENDOG localization in HeLa cells expressing HA‐KU80.
**Table S1.** Table of primary and secondary antibodies used in this study.
**Table S2.** siRNAs sequences used in this study.

## Data Availability

All the original data and information on reagents and protocols are available upon request. Correspondence and requests for materials should be addressed to Yonghwan Kim.
