# Supplementary material for: KU80 suppresses endonuclease G activity to preserve genomic integrity
Source: FEBS J. 2026 Mar 16;293(19):5952–66. doi: 10.1111/febs.70495 (PMC13628544; doi:10.1111/febs.70495)
Supplement: Supplementary file 1 [file FEBS-293-5952-s001.pdf]

Supplementary information for

KU80 Suppresses Endonuclease G Activity to Preserve Genomic Integrity

Jargalan Batsaikhan[1,6], Kwang-Hyun Park[2,5.6] Hae Ryung Chang[3,6], Eunyoung Jung[1,6], Myung-Jin Kim[1], Jeeyoon Kang[1], Hyunjo Shim[4,5], Eui-Jeon Woo[4,5,#], and Yonghwan Kim[1,#]

[1]Department of Biological Sciences and Research Institute of Women's Health, Sookmyung Women's University, Seoul 04310, Republic of Korea, [2]Critical Diseases Diagnostics Convergence Research Center, Korea Research Institute of Bioscience & Biotechnology (KRIBB), Daejeon 34141, Republic of Korea [3]Department of Life Science, Handong Global University, Pohang, Republic of Korea, [4]Biodesign & Editing Research Center, KRIBB, Daejeon 305-806, Republic of Korea, [5]Department of Proteome Structural Biology, KRIBB School of Bioscience, University of Science and Technology (UST), Daejeon 305-333, Republic of Korea, [6]These authors contributed equally to this work.

Supplementary Figure 1-4

Supplementary Table 1, 2

Supplementary Figure S1

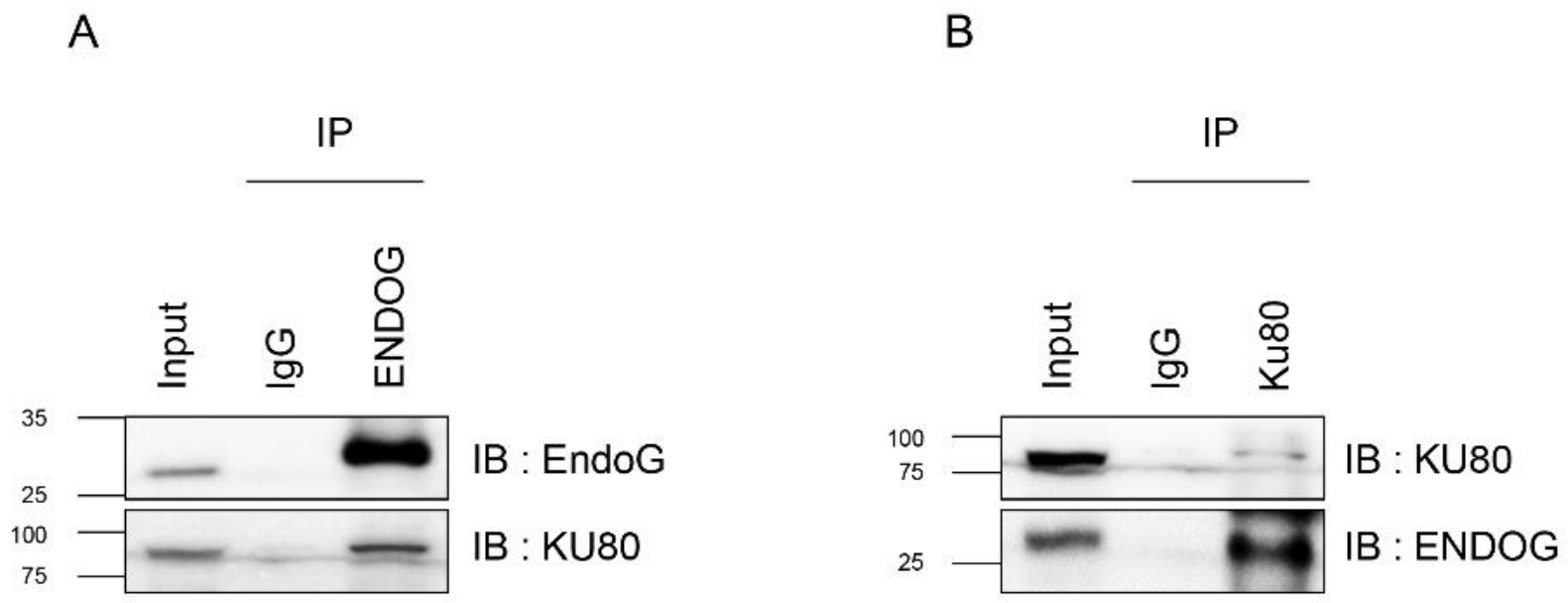

Supplementary Figure 1. Interaction between ENDOG and KU80 detected by endogenous immunoprecipitation in HeLa cells. (A) Endogenous ENDOG was immunoprecipitated, and co-precipitation of KU80 was examined by immunoblotting with the indicated antibody. **(B)** Reciprocal immunoprecipitation using KU80 antibody was performed, and the presence of ENDOG was detected by immunoblotting with the indicated andibody.

Supplementary Figure S2

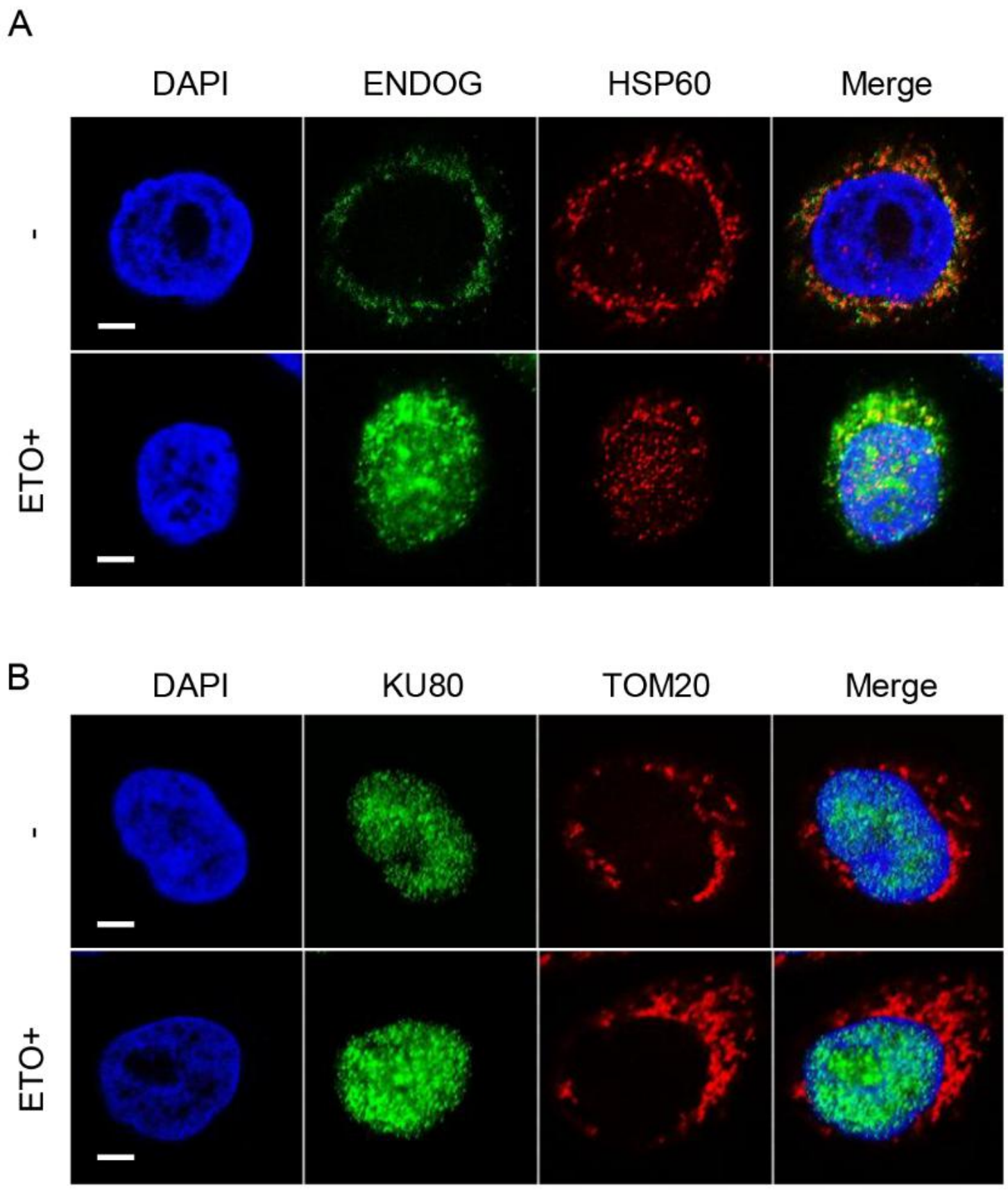

Supplementary Figure 2. Immunofluorescence analysis of ENDOG and KU80 localization in HeLa cells under untreated and etoposide-treated conditions. (A) Representative immunofluorescence images of HeLa cells stained for ENDOG (green) together with the mitochondrial marker HSP60 (red). Scale bars for all panels represent 5 ㎛. Before fixation, cells were treated with 50 μM etoposide for 16 h. **(B)** Representative immunofluorescence images of HeLa cells stained for KU80 (green) together with the mitochondrial marker TOM20 (red). Before fixation, cells were treated with 50 μM etoposide for 16 h.

Supplementary Figure S3

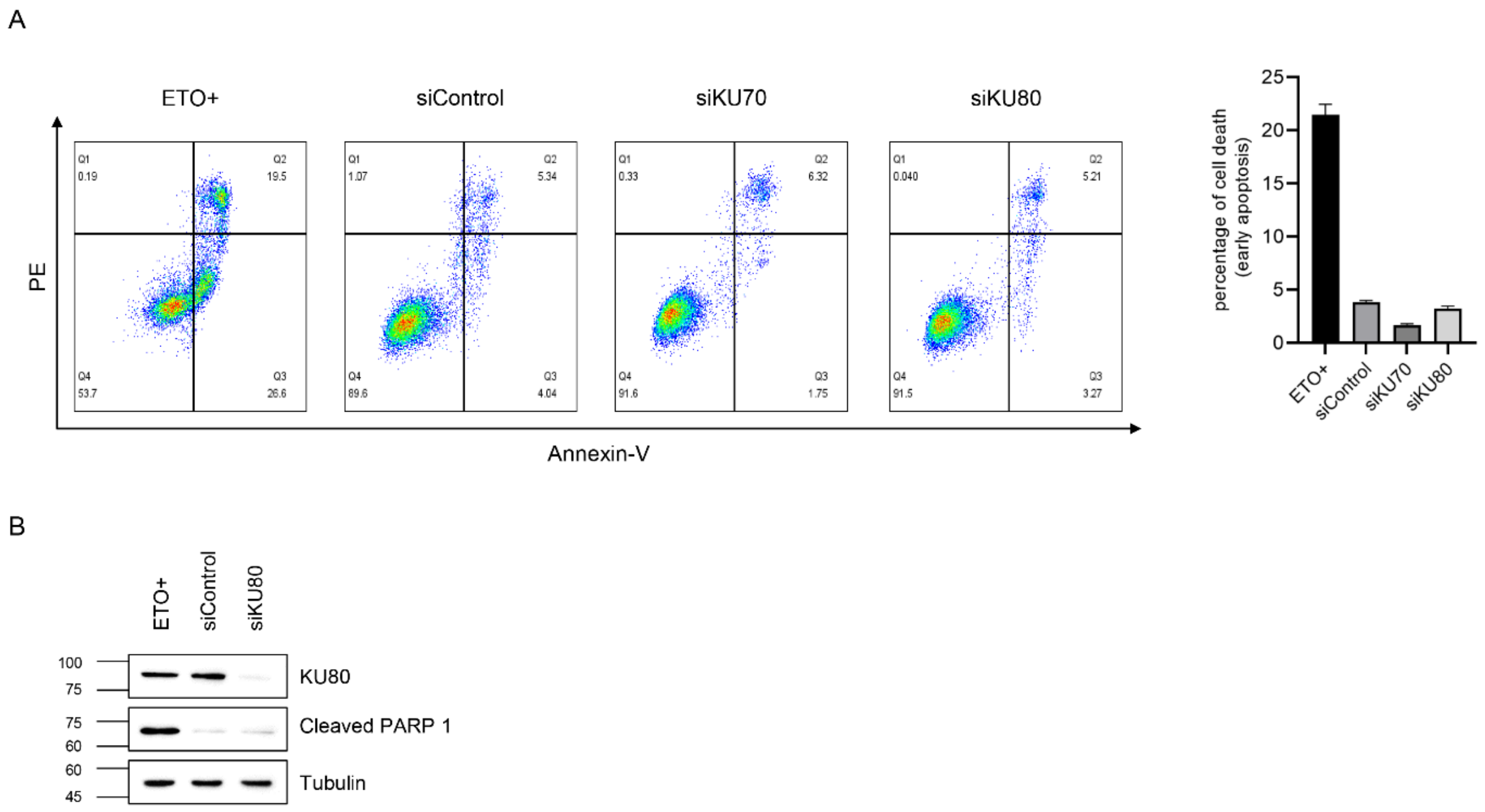

Supplementary Figure 3. Analysis of apoptotic induction following KU80 depletion. (A) Representative Annexin V/PI flow cytometry plots from HeLa cells transfected with siControl, siKU70, or siKU80 for 48 h. Before harvest, cells were treated with 1 μM etoposide for 16 h, and etoposide-treated cells were included as a positive control. Apoptotic populations were assessed based on Annexin V and PE staining (mean ± SD; n = 3 biologically independent replicates). **(B)** Western blot analysis of apoptotic markers (cleaved PARP1) in siControl- or siKU80-transfected HEK293T cells. Before harvest, cells were treated with 1 μM etoposide for 16 h. β-tubulin served as a loading control.

Supplementary Figure S4

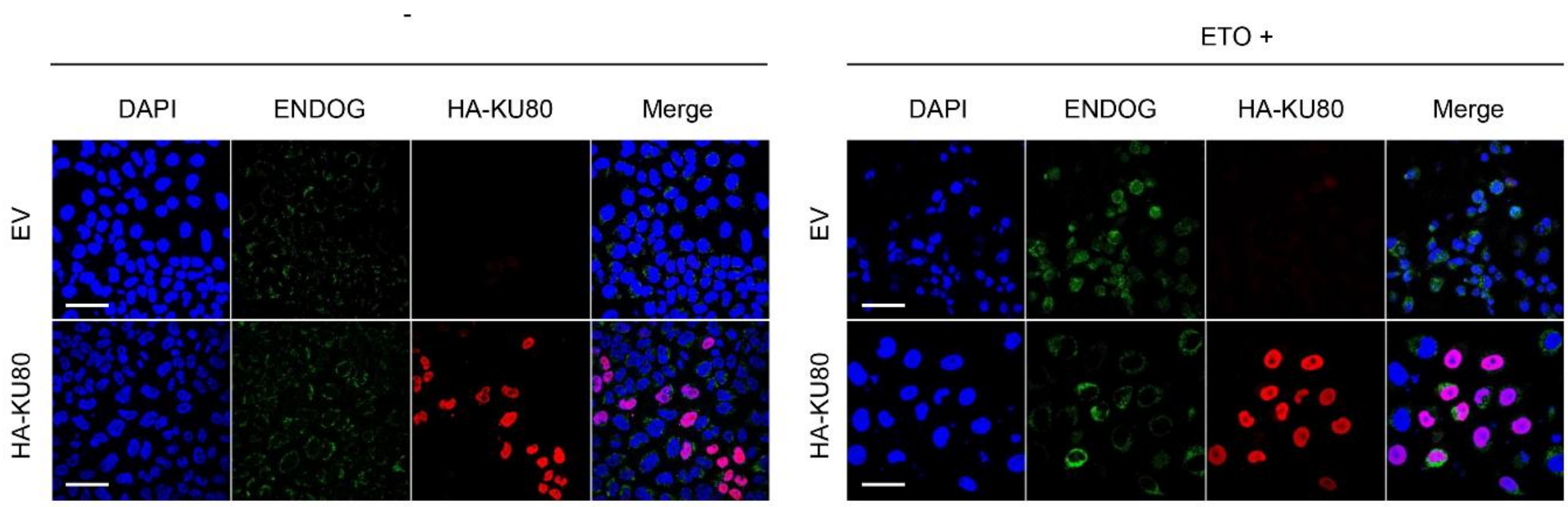

Supplementary Figure 4. Immunofluorescence analysis of ENDOG localization in HeLa cells expressing HA-KU80. Representative immunofluorescence images of HeLa cells stained for ENDOG (green) together with anti-HA antibody (red) (Scale bar, 50 ㎛). Cells were transiently transfected with either an empty vector (EV) or HA-KU80 plasmid. Before fixation, cells were treated with 50 μM etoposide for 16 h.

Supplementary Table 1. Table of primary and secondary antibodies used in this study.

**The table of the antibodies**					
**№**	**Name**	**Species**	**Distributer and catalog number**	**Dilution ratio**	**Dilution solution**
Primary antibodies					
1	EndoG	mouse	Santa Cruz, sc365359	IB 1:500<br>IF 1:500	3% BSA<br>1xPBG
2	KU80	mouse	Abcam, ab119935	IB 1:1000<br>IF 1:500	3% BSA<br>1xPBG
3	KU80	rabbit	Abcam, ab80592	IB 1:1000<br>IF 1:500	3% BSA<br>1xPBG
4	C-Myc	mouse	Santa Cruz, sc-40	IB 1:1000	3% BSA
5	HA	mouse	Bio Legend 901515	IB 1:1000	3% BSA
6	GAPDH	mouse	Santacruz, sc-47724	IB 1:1000	3% BSA
7	Actinin	mouse	Santa Cruz, sc17829	IB 1:1000	3% BSA
8	γH2AX	rabbit	Cell signaling, 9718S	IF 1:500	1xPBG
Secondary antibodies					
9	IgG light chain specific	mouse	Jackson Immuno Research	IB 1:2500	5% Skim milk
10	IgG light and heavy chain specific	mouse	Jackson Immuno Research	IB 1:2500	5% Skim milk

11	IgG light and heavy chain specific	rabbit	Jackson Immuno Research	IB 1:2500	5% Skim milk
12	IgG light and heavy chain specific 488	mouse	Abcam, ab150109	IF 1:2500	1xPBG
13	IgG light and heavy chain specific 594	mouse	Abcam, ab150108	IF 1:2500	1xPBG
14	IgG light and heavy chain specific 488	rabbit	Abcam, ab150061	IF 1:2500	1xPBG
15	IgG light and heavy chain specific 594	rabbit	Abcam, ab150064	IF 1:2500	1xPBG

*3% BSA: albumin bovine fraction and tween 20 in phosphate-buffered solution

*5% skim milk: skim milk powder and tween 20 in phosphate-buffered solution

*1xPBG: 0.5% albumin bovine fraction, 0.2% fish gelatin and phosphate-buffered solution in distilled water

Supplementary Table 2. Sequences of siRNAs used for gene silencing

The table of the siRNAs		
**Target gene**	**Target sequence**	
EndoG	1	AAGAGCCGCGAGTCGTACGTG
	2	CCAGAATGCCTGGAACAAC(TT)
	3	GGAACAACCTGGAGAAATATT
KU80	1	ACAAGGAUGAGAUUGCUUUdTsdT
	2	CAUGGAAAUCAAGUUCUAdTsdT
